# 

**Published:** 1920-01-03

**Authors:** 


### Medical Appointment.

ROYAL VICTORIA AND WEST HANTS HOSPITAL, BOURNEMOUTH.—Dr. A. C. Coles, M.D., D.Sc., M.R.C.P., F.R.S.E., of York House, Bournemouth, has been elected honorary physician. Mr. W. S. Richardson, F.R.C.S.Ed., of Melbury, Boscombe, has been elected honorary surgeon to succeed Mr. Bernard Scott, who retires after thirty years' service on the honorary staff, twenty-six of which have been spent as surgeon. Mr. Scott has been unanimously elected honorary consulting surgeon, and receives the very warm thanks of the Board for his splendid services to the hospital.

### Matrons' and Sisters' Appointments.

GROSVENOR HOSPITAL FOR WOMEN, S.W.—Miss Alice Thorne has been appointed matron. She was trained at the London Homœopathic Hospital, and has been assistant matron at the Chelsea Hospital for Women.

QUEEN ALEXANDRA'S MILITARY NURSING SERVICE FOR INDIA.—Miss M. Wilson and Miss C. Tracey Smith have been appointed nursing sisters.

POOR-LAW INFIRMARY, CHELMSFORD.—Miss Elizabeth Annie Richardson has been appointed superintendent nurse. She was trained at Mile End Infirmary, and has been superintendent nurse at Pontefract Union Infirmary.

DISABLED SAILORS' AND SOLDIERS' HOME, BROUGHTON HOUSE, MANCHESTER.—Miss Mary Tracey has been appointed matron. She was trained at the Royal Infirmary, Liverpool, and has been matron of Sedgeley Park V.A.D. Hospital, Manchester.

PUBLIC HOSPITAL, DEMERARA.—Miss Susie Clapham has been appointed matron. She was trained at the Royal Hospital, Portsmouth, and at the City of London Lying-in Hospital, and has been matron of the Sanatorium, Silver City, New Mexico, and of the Civil Hospital, Gibraltar.

SCAFFOLD HILL INFECTIOUS DISEASES HOSPITAL, NORTH SHIELDS.—Miss Emily L. Ross has been appointed nurse-matron. She was trained at Hampstead General Hospital, where she was later sister; she has been pupil-housekeeper and night sister at the South Devon and East Cornwall Hospital, Plymouth, assistant matron at Tiverton Hospital, North Devon, matron of the Pontypridd Cottage Hospital, of the Ear and Throat Hospital, Birmingham, and sister and assistant matron of the City Isolation Hospital, Worcester.

MUNICIPAL MATERNITY HOME, LEWISHAM.—Miss Elsa Marguerite Murby has been appointed matron. She was trained at Charing Cross Hospital, and was later sister there.

COTTAGE HOSPITAL, LYNTON.—Miss L. Wilkinson has been appointed sister-in-charge. She was trained at the Royal Cornwall Infirmary, Truro, and has since been matron of Accrington Red Cross Hospital.

UNION INFIRMARY, COVENTRY.—Miss Hannah M. Newton has been appointed superintendent nurse. She was trained at Leeds Township Infirmary, and has since been sister and assistant matron of Northumberland War Hospital, Newcastle-on-Tyne; sister at Kendray Hospital, Barnsley; and assistant superintendent of nurses in Sunderland.

INFECTIOUS DISEASES HOSPITAL, KNARESBOROUGH, AND THE SMALLPOX HOSPITAL, KILLINGHALL.—Miss Ada Copeland has been appointed matron. She was trained at Edinburgh Royal Infirmary and at the Darlington Fever Hospital, and has been matron of the Isolation Hospitals at Acomb, York, Chichester, and Bishop's Stortford, respectively.

ROYAL WATERLOO HOSPITAL, S.E.—Miss E. A. Batty has been appointed sister. She was trained at the Essex County Hospital, Colchester, has been ward and theatre sister there, has held a post at Queen Charlotte's Hospital, and has served in Queen Alexandra's Imperial Military Nursing Service Reserve.

COTTAGE HOSPITAL, CLONMEL.—Miss Ethel Benning has been appointed matron. She was trained at the Richmond, Whitworth, and Hardwick Hospitals, Dublin.

HOSPITAL FOR INFECTIOUS DISEASES, WEST HEATH, CONGLETON.—Miss F. E. Bates has been appointed matron. She was trained at St. Mary's Hospital, Paddington, and has done Army nursing.

COTTAGE HOSPITAL, BALLYMENA, CO. ANTRIM.—Miss Agnes Wilson has been appointed matron. She was trained at the Royal Victoria Hospital, Belfast, and has been sister-in-charge of the Children's Convalescent Home, Carrickfergus.

LADY MARGARET HOSPITAL, BROMLEY, KENT.—Miss Winifred H. Siddle has been appointed sister-in-charge. She was trained at the Royal Victoria Hospital, Newcastle-on-Tyne, where she was later sister.

LADY MARGARET HOSPITAL (PAYING WARDS), UPPER TULSE HILL, S.W. 2.—Miss Margaret Careswell has been appointed sister-in-charge. She was trained at the General Hospital, Forfar, where she was later sister and temporary matron. She has also been sister at the Hospital St. Cross, Rugby, and of the British Red Cross Society, and matron of the Uckfield Cottage Hospital.

CHILD WELFARE CENTRE, MANCHESTER.—Miss Jessie Emsley has been appointed assistant superintendent. She was trained at the Liverpool Royal Infirmary, and has since been sister at the East Suffolk Hospital, Ipswich, the Beckett Hospital, Barnsley, and at the Stanley Hospital, Liverpool. Miss Emsley has also seen service in France with the St. John Ambulance Brigade Hospital and with Q.A.R.N.N.S.R. at Plymouth. She holds the certificate of the Central Midwives Board, is a member of the College of Nursing, and an honorary serving sister of the Order of St. John of Jerusalem.

LONDON HOMŒOPATHIC HOSPITAL, GREAT ORMOND STREET, W.C.—Miss Clara Robinson, R.R.C., has been appointed matron, *vice* Miss Edith Annie Eddison, R.R.C., resigned. Miss Robinson was trained at St. Thomas's Hospital, has been sister at Portsmouth Royal Hospital and Charing Cross Hospital, and assistant matron at the London Homœopathic Hospital. She has been matron of the Buchanan Hospital, St. Leonards-on-Sea, and has been twice mentioned in despatches.

THE HOSPITAL, NORTH LONSDALE, BARROW-IN-FURNESS.—Miss Constance Passmore has been appointed sister. She was trained at the Victoria Hospital, Barnet, has been masseuse at the 2nd Northern General Hospital under the Almeric Paget Military Massage Corps, sister at the West Suffolk Hospital, Bury St. Edmunds, and temporary sister at the West London Hospital, Hammersmith. Miss Passmore holds the certificate of the Incorporated Society of Trained Masseuses.

### THE COLLEGE OF NURSING (Limited by Guarantee).

### THE LATEST DEVELOPMENT.

### A COLLEGE QUARTERLY BULLETIN.

We learn that the Council of the College of Nursing has decided to publish a quarterly bulletin, the first four numbers of which will be circulated free to all members. The object of the bulletin will be to keep members of the College informed upon matters affecting the nursing profession as a whole, upon the policy of the Council, and upon the proceedings of Local Centres. A section will also be devoted to hospital and nursing news, to articles on the different branches of nursing, and to reviews of medical and nursing publications. With the first issue of the bulletin in about a month's time it is proposed to circulate nomination papers for the Council election next June.

### LONDON CENTRE.

TUESDAY, JANUARY 6, 4.30 P.M.—Meeting of the Executive Committee in the Club Room at 7 Henrietta Street, Cavendish Square.

THURSDAY, JANUARY 8, 8 P.M.—The first of the lectures, arranged for the spring session, will be given by Miss Bagley, Head of the Polytechnic School of Speech Training, in the Rooms of the Medical Society, Chandos Street, Cavendish Square, W. 1. The subject will be "Public Speaking: the Voice, its Possibilities, and Powers." The public are admitted on payment of 6d. at the door; members on presentation of the membership card.

It is proposed to hold a Members' Meeting in January or early February; members who wish to have subjects included on the agenda for that meeting are asked to communicate with the Hon. Secretary before January 16.

